# Study protocol of a phase II clinical trial (KSCC1501A) examining oxaliplatin + S-1 for treatment of HER2-negative advanced/recurrent gastric cancer previously untreated with chemotherapy

**DOI:** 10.1186/s12885-017-3937-6

**Published:** 2018-01-08

**Authors:** Hiroshi Saeki, Yasunori Emi, Eiji Oki, Shoji Tokunaga, Yoshihiro Kakeji, Yoshito Akagi, Hideo Baba, Eishi Baba, Yoshihiko Maehara, Kyushu Study group of Clinical Cancer (KSCC)

**Affiliations:** 1 Department of Surgery and Science, Graduate School of Medical Sciences, Kyushu University, 3-1-1 Maidashi, Higashi-ku, Fukuoka, 812-8582 Japan; 2 Department of Surgery, Saiseikai Fukuoka General Hospital, Fukuoka, Japan; 3 Medical Information Center, Kyushu University Hospital, Fukuoka, Japan; 4 Division of Gastrointestinal Surgery, Department of Surgery, Graduate School of Medicine, Kobe University, Kobe, Japan; 5 Department of Surgery, Kurume University School of Medicine, Fukuoka, Japan; 6 Department of Gastroenterological Surgery, Graduate School of Medical Sciences, Kumamoto University, Kumamoto, Japan; 7 Department of Comprehensive Clinical Oncology, Faculty of Medical Sciences, Kyushu University, Fukuoka, Japan

**Keywords:** Metastatic gastric cancer, First-line chemotherapy, Oxaliplatin, S-1, Japanese patients

## Abstract

**Background:**

Oxaliplatin + S-1 is a recognized treatment regimen in Japan, but there are no Japanese clinical data on an oxaliplatin dose of 130 mg/m^2^. The current research involves a single-arm, prospective, phase II clinical trial to examine the efficacy and safety of oxaliplatin + S-1 with an oxaliplatin dose of 130 mg/m^2^ to treat HER2-negative advanced/recurrent gastric cancer previously untreated with chemotherapy in Japan.

**Methods/design:**

The primary endpoint of this trial will be the response rate, and the secondary endpoints will be the safety profile of oxaliplatin + S-1, progression-free survival, the response rate in subjects under the age of 75, overall survival, time to treatment failure, duration of treatment, time to failure of strategy, and dose intensity. The threshold response rate is 45% and the expected response rate is 60%. Assuming that a one-tailed score test will be performed with an α of 0.05, 68 patients are needed to ensure a statistical power of 80%. Planned enrollment is 70 subjects and the total duration of this trial is expected to be 3 years.

**Discussion:**

Since replacing cisplatin with oxaliplatin should provide the same level of therapeutic efficacy while limiting adverse events and simplifying treatment, oxaliplatin + S-1 may be increasingly used to treat gastric cancer in Japan. Verifying the efficacy and safety of oxaliplatin + S-1 with an oxaliplatin dose of 130 mg is an important task that the current trial has set out to achieve.

**Trial registration:**

The protocol was registered at the website of the University Hospital Medical Information Network (UMIN), Japan (protocol ID UMIN000017550) on May 29, 2015. The details are available at the following web address: http://www.umin.ac.jp/ctr/.

## Background

Based on outcomes of domestic and foreign clinical trials, the median survival time (MST) for patients with gastric cancer is 6–13 months [1–4]. The standard chemotherapy for advanced/recurrent gastric cancer combines fluorinated pyrimidines and platinum-based drugs. Trastuzumab is a monoclonal antibody that is used to treat human epidermal growth factor receptor 2 (HER2)-positive gastric cancer [5, 6]. Since the results of the SPIRITS study [2] were reported in 2007, cisplatin + S-1 (SP) has served as the standard therapy for advanced/recurrent gastric cancer. However, studies overseas have reported that cisplatin can be replaced with oxaliplatin as the platinum-based drug used in combination with S-1. Replacing cisplatin with oxaliplatin should provide the same level of therapeutic efficacy while limiting adverse events and simplifying treatment. The REAL-2 study is a non-inferiority study that compared a therapy combining epirubicin with capecitabine or 5-FU and a therapy combining epirubicin with oxaliplatin or cisplatin in terms of overall survival (OS) [7]. The REAL-2 study indicated that 5-FU can be replaced with capecitabine and that cisplatin can be replaced with oxaliplatin. The German Cancer Society (Arbeitsgemeinschaft Internistische Onkologie, or AIO) conducted a comparative study of 5-FU/leucovorin + oxaliplatin and 5-FU/leucovorin + cisplatin [8]. The study reported that 5-FU/leucovorin + oxaliplatin resulted in a better progression-free survival (PFS) and OS (although not significantly better) than 5-FU/leucovorin + cisplatin and that 5-FU/leucovorin + oxaliplatin reduced adverse events besides peripheral sensory neuropathy. Thus, the study’s results suggested that replacing cisplatin with oxaliplatin might prove useful. Based on these findings, the National Comprehensive Cancer Network (NCCN) Guidelines, Version 1. 2014 and the ESMO-ESSO-ESTRO Clinical Practice Guidelines list a regimen including oxaliplatin as a recommended regimen.

In Japan, oxaliplatin + S-1 (SOX) (oxaliplatin dose: 100 mg/m^2^) and SP were compared in the G-SOX study, but SOX did not demonstrate non-inferiority in comparison to SP [9]. A recent application sought approval of oxaliplatin based on data in the public domain [in Japan, this system is used to approve off-label use of an approved drug without requiring clinical trials]. As a result, use of oxaliplatin in the treatment of gastric cancer is now covered by national health insurance. However, the data that served as the basis for this decision were from foreign clinical trials [7, 8], so the dose of oxaliplatin to treat gastric cancer is 130 mg/m^2^. The Japanese Gastric Cancer Association has recognized SOX (oxaliplatin dose: 130 mg/m^2^) as a regimen for treatment of gastric cancer. In Japan, SOX will presumably be used more than SP in the future in order to limit adverse events and simplify treatment. Although the G-SOX study compiled clinical data on SOX with an oxaliplatin dose of 100 mg/m^2^, there are no Japanese clinical data on SOX with an oxaliplatin dose of 130 mg/m^2^ for treatment of advanced/recurrent gastric cancer.

Thus, the current trial is the first in Japan to compile clinical data on SOX with an oxaliplatin dose of 130 mg/m^2^. The results obtained will presumably facilitate the proper use of oxaliplatin (130 mg/m^2^) + S-1 to treat HER2-negative advanced/recurrent gastric cancer previously untreated with chemotherapy. In a phase I/II study of first-line therapy for patients with metastatic colorectal cancers that was conducted in Japan, SOX with an oxaliplatin dose of 130 mg/m^2^ has previously been evaluated, and found to be easily manageable [10]. Based on this evidence, we aimed to conduct a phase II clinical trial that would examine the efficacy and safety of SOX with an oxaliplatin dose of 130 mg/m^2^ for advanced/recurrent gastric cancers, without using a phase I study.

## Methods/Design

### Study setting

There are no prospective clinical trials on SOX with an oxaliplatin dose of 130 mg/m^2^ to treat advanced/recurrent gastric cancer in Japan, so a single-arm, prospective phase II clinical trial will be conducted to verify the efficacy and safety of that therapy. This trial will provide reference data for the future. SOX with an oxaliplatin dose of 130 mg/m^2^ is already a primary therapy option for advanced/recurrent gastric cancer, so a confirmatory phase III trial is not planned.

### Endpoints

The primary endpoint for the current trial will be the response rate, and the response rate will be determined in accordance with RECIST v1.1. Like the G-SOX study, this trial will use eligibility criteria that include target lesions (measurable lesions) documented at the baseline in accordance with RECIST v1.1. Trial subjects will be patients with target lesions to allow comparison of efficacy between studies. The secondary endpoints will be the safety profile of SOX, PFS, the response rate in subjects under the age of 75, OS, time to treatment failure, duration of treatment, time to failure of strategy, and dose intensity. Particular attention will be paid to thrombocytopenia, peripheral sensory neuropathy, and allergic reactions.

### Eligibility criteria and exclusion criteria

The patients with advanced/recurrent gastric cancers who are not amenable to curative surgery are eligible for enrollment. Patients in whom the center of the primary tumor is located above the gastroesophageal junction are excluded from enrollment in this study. Gastric cancer is staged according to the Japanese Classification of Gastric Carcinoma -14th Japanese version which corresponds to Third English edition [11]. Eligibility and exclusion criteria for this trial are listed in Tables 1 and 2, respectively.

**Table 1:** Eligibility criteria

1) The individual consents in writing to receipt of the protocol treatment. 2) A lead investigator deems that the patient can be treated with the protocol. 3) The cancer is histopathologically confirmed (via resected surgical specimens or biopsied tissue) to be a common type of gastric cancer (adenocarcinoma). 4) Up to 28 days prior to enrollment, contrast-enhanced CT scans of the trunk confirm that 1 or more target lesions is present according to RECIST v1.1 (measurable lesions according to RECIST v1.1). 5) The primary tumor or a metastatic focus is determined to be HER2-negative. Negativity for HER2: 0–1+ on an ICH test, or 2+ on an IHC test and negative on a FISH test (If the result of the IHC test is 2+, the result of the FISH test must be negative, i.e. no HER2 gene amplification). 6) The patient has not previously received chemotherapy, immunotherapy, or radiation therapy (except for local irradiation of bone metastases)^*^. 7) The patient has advanced/recurrent gastric cancer not amenable to curative surgery. 8) When a patient undergoes surgery for gastric cancer in the form of extensive surgery, standard surgery, or some other procedure, trial participation will be at least 2 weeks afterwards when postoperative complications have abated. 9) The patient’s age upon enrollment is over 20 and under 80 years. 10) The patient has a PS of 0 or 1 on the ECOG Scale. 11) The patient is readily able to take medication orally (patients requiring hyperalimentation for nutritional support are not eligible). 12) The patient is fully expected to survive for 3 months or longer from the day of enrollment. 13) The patient has no severe dysfunction of major organs (bone marrow, liver, kidneys, heart, lungs, etc.) and the patient’s laboratory results from up to 14 days prior to enrollment fall within the criteria. A patient may not receive a transfusion, a hematopoietic growth factor, or a blood product up to 14 days prior to the day that laboratory results are obtained. i) WBC count ≤12,000 /mm^3^ ii) Neutrophil count ≥1500 /mm^3^ iii) Platelet count ≥100,000 /mm^3^ iv) Hemoglobin ≥8.0 g/dL v) Total bilirubin ≤1.5 mg/dL vi) AST,ALT ≤100 IU/L vii) AST,ALT, 200 IU/L or lower when liver metastasis is noted viii) Albumin ≥2.5 g/dL ix) Serum creatinine ≤1.5 mg/dL x) Creatinine clearance ≥60 mL/min	

^*^Of potential subjects who have received adjuvant chemotherapy with S-1 alone, only individuals who are found to have recurrence at least 180 days after the conclusion of adjuvant chemotherapy will be eligible for enrollment. Patients with recurrence who have received adjuvant chemotherapy with a drug other than S-1 or perioperative adjuvant chemotherapy with S-1 + α will not be eligible for enrollment, regardless of the duration of the period prior to recurrence

**Table 2:** Exclusion criteria

1) A patient who has received a transfusion, blood product, or hematopoietic growth factor such as G-CSF up to 14 days prior to enrollment. 2) The patient has severe drug hypersensitivity (particularly to platinum analogs, 5-FU, or S-1). 3) The patient has peripheral neuropathy affecting the sensory nerves (Grade 1 or worse). 4) The patient has an active infection. 5) The patient has poorly controlled hypertension. 6) The patient has poorly controlled diabetes. 7) The patient has heart disease that may pose a problem. 8) The patient has severe pulmonary disease. 9) The patient has a psychiatric disorder that may pose a problem or a history of central nervous system dysfunction. 10) The patient has active gastrointestinal tract bleeding requiring repeated transfusions. 11) The patient is receiving phenytoin, warfarin potassium, or flucytosine. 12) The patient has moderate or more severe fluid accumulation in body cavities, such as pleural effusions and ascites, that continually requires treatment such as drainage. 13) The patient has brain metastasis or clinical features suggesting brain metastasis. 14) The patient has extensive bone metastasis (as determined by the patient’s primary physician). 15) The patient has watery diarrhea (watery stool) (Grade 2 or worse). 16) The patient has active multiple cancers. 17) The patient has a history of receiving platinum-based antineoplastic agents (e.g. oxaliplatin and cisplatin). 18) A woman who is pregnant, nursing, or possibly pregnant or a man who is trying to conceive with a partner. 19) The patient tests positive for HBsAg or antibodies to HCV. 20) A patient who is deemed to need antiviral therapy for HBV-related hepatitis. 21) A lead investigator or the patient’s primary physician otherwise deems that participation in this trial is not appropriate for the patient.	

### Treatment

A course of treatment will last 21 days, and the course will be repeated until disease progression or intolerable toxicity is observed. On day 1, oxaliplatin 130 mg/m^2^ will be administered intravenously. In accordance with body surface area, S-1 40–60 mg will be given orally twice a day, once after breakfast and once after dinner, for 14 consecutive days, followed by a 7-day rest period.

If the patients exhibit toxicities that meet the reduction criteria, the oxaliplatin dose of 130 mg/m^2^ will be gradually reduced to 100 mg/m^2^, 75 mg/m^2^, and 50 mg/m^2^. If further reductions are required, the administration of oxaliplatin will be stopped. If Grade 2 peripheral sensory neuropathy is evident, the oxaliplatin dose will be reduced. If Grade 3 peripheral sensory neuropathy is evident, the administration of oxaliplatin in that course will be foregone and therapy began with S-1 alone. If the patient’s platelet count is lower than 75,000 /mm^3^, the oxaliplatin dose will be reduced. If administration of oxaliplatin is foregone or the oxaliplatin dose has to be reduced beyond the specified range, only administration of oxaliplatin will be discontinued but administration of S-1 alone will continue. Such treatment is still considered to be treatment within the protocol. However, foregoing administration of S-1 and administering oxaliplatin alone is not permitted. If the dose of S-1 or the doses of oxaliplatin and S-1 are reduced beyond the specified range, the protocol treatment will be discontinued (i.e. a decision to continue treatment will not be made).

### Follow-up

Lesions will be measured with contrast-enhanced CT scans of the trunk every 6 weeks. All subjects will continue to undergo imaging unless disease progression is noted. If disease progression is not noted but the protocol treatment is discontinued due to adverse events, imaging will continue to be performed until “disease progression” is verified.

### Sample size and follow-up period

Planned enrollment will be 70 subjects. The duration of patient recruitment will be 1 year, and the follow-up period will be 2 years from final patient enrollment. The total duration of this trial will be 3 years.

### Statistical considerations

SP is a standard chemotherapy for advanced/recurrent gastric cancer previously untreated with chemotherapy. The response rate to this form of chemotherapy was 54% [95% CI: 46–62] in the SPIRITS study [2]. In contrast, the response rate to SOX (oxaliplatin dose: 100 mg/m^2^) was 56% [95% CI: 50–62] in the G-SOX phase III study [9].

Based on the lower bound of the 95% CI for the response rate to SP in the SPIRITS study and to SOX in the G-SOX phase II and phase III studies (oxaliplatin dose: 100 mg/m^2^), the threshold response rate in the current trial is 45%. Based on the upper bound of the 95% CI for the response rate to SP in the SPIRITS study and to SOX in the G-SOX phase II and phase III studies (oxaliplatin dose: 100 mg/m^2^) and based on the response rate to SOX (oxaliplatin dose: 100 mg/m^2^) in the G-SOX phase II study (59%), the expected response rate in the current trial is 60% since the protocol treatment was SOX with an oxaliplatin dose of 130 mg/m^2^. In the current trial, the threshold response rate is 45% and the expected response rate is 60%. Assuming to perform the one-tailed score test with an α of 0.05, 68 patients are needed to ensure the statistical power of 80%. Given the potential for drop-outs and patients who become ineligible after enrollment, target enrollment is 70 patients.

### Study organization

The Kyushu Study group of Clinical Cancer (KSCC) is responsible for the project management of the trial. The study was organized by the Clinical Research Support center Kyushu (CReS-Kyushu), Fukuoka, Japan, and the tasks of CReS-Kyushu include the coordination of investigators’ meetings, monitoring, data management, and audits.

### Data management, control of data consistency, quality control, monitoring and audits

The investigator or a designated representative must enter all information required by the protocol in the electronic case report form. Any entry and correction in the Remote Data Entry System will be recorded automatically. Automatic checks for data completeness, validity, and consistency will be programmed by CReS-Kyushu. Clinical central monitoring will be performed by the Coordination Centre of CReS-Kyushu. Monitoring procedures will be adapted to the study-specific risks for patients. Standard operating procedures (SOP) will be interpreted to ensure patient safety and the integrity of the clinical data, e.g. the primary endpoint complies with the study protocol, and queries will be generated. The investigator or a designated representative is obliged to provide clarification or respond to queries. If no further corrections are to be made in the database, it will be locked and used for statistical analysis. All data management procedures will be carried out according to the current SOPs of CReS-Kyushu. Regular on-site monitoring visits are not planned, but KSCC group rules will be regularly audited during this study.

### Participating institutions

Kyushu University Hospital, National Kyushu Medical Center, Kyushu Cancer Center, Kyushu Central Hospital, Saiseikai Fukuoka General Hospital, Fukuoka City Hospital, Fukuoka Dental College Medical & Dental Hospital, Saiseikai Yahata General Hospital, Steel Memorial Yawata Hospital, Japan Community Health Care Organization Kyushu Hospital, Fukuoka Higashi Medical Center, Iizuka Hospital, Munakata Medical Association Hospital, Tagawa Municipal Hospital, Social Insurance Tagawa Hospital, Tanushimaru Center Hospital, Kurume University Hospital, Saga University Hospital, Ureshino Medical Center, Japan community Health care Organization Isahaya General Hospital, Sasebo City General Hospital, Sasebo Chuo Hospital, Nagasaki University Hospital, Kouseikai Hospital, Kakizoe Hospital, Saiseikai Kumamoto Hospital, Kumamoto Medical Center, Kumamoto University Hospital, Japan community Health care Organization Hitoyoshi Medical Center, Japan community Health care Organization Kumamoto General Hospital, Amakusa Medical Center, Oita Prefectural Hospital, Oita Red Cross Hospital, Oita Medical Center, Kyushu University Beppu Hospital, Beppu Medical Center, Oita University Hospital, Nakatsu Municipal Hospital, Minami Kyushu National Hospital, Imakiire General Hospital, Kagoshima University Medical And Dental Hospital, Kagoshima Kouseiren Hospital, Imamura Hospital, Izumi Regional Medical Center, Heart Life Hospital, Chubu Tokushukai Hospital, Naha City Hospital, University of the Ryukyus Hospital, Tomishiro Central Hospital, Hiroshima Prefectural Hospital, Okayama Rousai Hospital, Matsuyama Red Cross Hospital, Kagawa University Hospital, Kobe City Medical Center General Hospital, Kobe University Hospital, Hyogo Prefectural Kakogawa Medical Center, Sano Hospital, Yokkaichi Municipal Hospital, Tokai Central Hospital, Gifu Prefectural Tajimi Hospital, Nakatsugawa Municipal General Hospital, Gifu University Hospital, Toyohashi Medical Center, Tosei General Hospital, Ichinomiya Municipal Hospital, Nagoya University Hospital, Komaki City Hospital, Konan Kosei Hospital, Aichi Cancer Center Aichi Hospital, Kainan Hospital, Atsumi Hospital, Aichi Medical University Hospital, Nagoya Medical Center, Aizawa Hospital, Dokkyo Medical University Hospital, Misawa City Hospital, Aomori City Hospital, Hokkaido University Hospital, Sapporo-Kosei General Hospital.

## Discussion

The primary endpoints in the G-SOX study were a PFS with a non-inferiority margin of 1.30 and an OS with a non-inferiority margin of 1.15 [9]. Domestic phase I/II trials of oxaliplatin to treat colon cancer used a recommended dose of 130 mg/m^2^, much like foreign trials used, but Grade 3 or worse thrombocytopenia appeared at a high rate (27%) [10]. In addition, thrombocytopenia due to bleeding from the primary tumor may be more prevalent in gastric cancer than in colon cancer. Given these facts, thrombocytopenia could result in a lower dose intensity of S-1 (a key drug), so the G-SOX study used an oxaliplatin dose of 100 mg/m^2^. The G-SOX study enrolled 685 patients, 342 of whom received SP and 343 of whom received SOX. Patients receiving SP had a PFS of 5.4 months while patients receiving SOX had a PFS of 5.5 months (*p* = 0.0044, HR: 1.004, 95% CI: 0.840–1.199). Patients receiving SP had an OS of 13.1 months while patients receiving SOX had an OS of 14.1 months (*p* = 0.0583, HR: 0.969, 95% CI: 0.812–1.157). Results of the G-SOX study demonstrated that SOX was not inferior to SP in terms of PFS, but results failed to demonstrate that SOX was not inferior to SP in terms of OS. Patients receiving SOX had significantly fewer adverse events that were Grade 3 or worse (74.9% for patients receiving SP vs. 63.3% for patients receiving SOX, *p* = 0.0011), and patients receiving SOX were hospitalized for significantly less time with each course of treatment (6.00 days for patients receiving SP vs. 0.83 day for patients receiving SOX, *p* < 0.0001) [9]. Use of oxaliplatin to treat gastric cancer is covered by national health insurance, but the dose is 130 mg/m^2^. This dose was decided on the basis of data from foreign clinical trials [7, 8]. However, there are no clinical data on use of SOX with an oxaliplatin dose of 130 mg/m^2^ to treat unresectable gastric cancer in Japan. Thus, the current trial is the first to compile such data, and findings should facilitate the proper use of SOX to treat gastric cancer. Hironaka et al. have reported the results of a randomized phase II trial that compared the combinations of S-1 with leucovorin only, with leucovorin and oxaliplatin, and with cisplatin, respectively, in patients with advanced gastric cancers [12]. The objective response rate in the group that underwent treatment with S-1 plus leucovorin and oxaliplatin (with an oxaliplatin dose of 85 mg/m^2^, once, every 2 weeks) was 66%, and the authors concluded that an S-1 plus leucovorin and oxaliplatin regimen was more active than other regimens, and had acceptable toxic effects. Although the protocol of this regimen seems promising, an oxaliplatin dose of 85 mg/m^2^, once, every 2 weeks is not approved by the National Health Insurance. Therefore, this regimen was not used in our study, because our current study was conducted in an investigator-initiated fashion. A phase III trial comparing S-1 plus leucovorin and oxaliplatin with S-1 plus cisplatin as chemotherapy for patients with advanced gastric cancers is currently ongoing (NCT02322593).

In the current trial, the response rate (the primary endpoint) or PFS (a secondary endpoint) can serve as a surrogate endpoint for OS. A study of advanced/recurrent gastric cancer examined whether PFS could serve as a surrogate endpoint for OS, and results indicated that the two are moderately correlated [13]. The study concluded that the question of whether or not the response rate or PFS could serve as a surrogate endpoint would need to be studied further. In the current trial, the response rate will serve as the primary endpoint for convenience. This approach will allow objective assessment of efficacy. If the response rate is the primary endpoint, then measurable lesions must be determined in accordance with RECIST v1.1 at the baseline. When unresectable advanced, recurrent, or metastatic gastric cancer is encountered in clinical practice, it is mostly disseminated throughout the peritoneal cavity in half or more patients. The current trial requires subjects to have target lesions, so the assembled sample may differ slightly from patients with gastric cancer who are encountered in clinical practice. One aim of this trial is to allow comparison to the G-SOX study, so subjects must have target lesions as they did in the G-SOX study.

Chemotherapy with trastuzumab is the standard treatment for HER2-positive gastric cancer, so investigators were obliged to determine the HER2 status of potential subjects prior to their enrollment in the current trial. Actual subjects were limited to patients with HER2-negative gastric cancer. According to the JFMC44–1101 study (an observational study of the HER2 positive rate in patients with advanced/recurrent gastric cancer that is not amenable to curative surgery) [14], the HER2 positive rate in Japan is 20.5% (293/1427). An analysis solely of Japanese subjects in the ToGA trial [5] indicated that the HER2 positive rate was 20.0% (82/409). In the HERBIS-1A trial (a phase II trial of S-1 + cisplatin + trastuzumab for treatment of patients with HER2-positive gastric cancer (IHC 3+ or IHC 2+ and FISH positive) that was conducted in Japan [15], 83% of subjects (45/54) had an IHC score of 3+ while 17% (9/54) had an IHC score of 2+. Thus, the cancers treated in the current trial represent about 80% of all gastric cancers in terms of HER2 status.

Standard adjuvant chemotherapy for treatment of Stage II or III gastric cancer is to administer S-1 alone for 1 year in Japan. An observational study assessed the efficacy of SP in terms of the period from receipt of S-1 as adjuvant chemotherapy until recurrence. When cancer recurred within 6 months, SP resulted in a PFS of 2.3 months and an MST of 7.3 months. When cancer recurred after 6 months, SP resulted in a PFS of 6.2 months and an MST of 16.6 months [16]. Cancer that recurred while a patient was receiving S-1 as adjuvant chemotherapy or within 6 months of the final round of S-1 was deemed to be refractory to S-1, but cancer that recurred after 6 months was deemed to be responsive even if the patient was receiving S-1 again. Accordingly, patients with cancer that responded to S-1 have been deemed eligible for enrollment in the current trial.

Cisplatin can be replaced with oxaliplatin as the platinum-based drug used in combination with S-1. Replacing cisplatin with oxaliplatin should provide the same level of therapeutic efficacy while limiting adverse events and simplifying treatment by not requiring hydration. Thus, SOX may be increasingly used to treat gastric cancer in Japan. Verifying the efficacy and safety of SOX with an oxaliplatin dose of 130 mg is an important task that the current trial has set out to achieve. If this current phase II study meets the defined endpoints, we further plan to perform a prospective controlled study that would compare the safety and efficacies of SOX regimens with oxaliplatin doses of 100 mg/m^2^ and 130 mg/m^2^, respectively.

## Abbreviations

CT: computed tomography
ESMO-ESSO-ESTRO: European Society for Medical Oncology-European Society of Surgical Oncology-European Society for Radiotherapy & Oncology
RECIST: Response Evaluation Criteria in Solid Tumors
UMIN: University Hospital Medical Information Network
